# Effect of Acetylation of Two Cellulose Nanocrystal Polymorphs on Processibility and Physical Properties of Polylactide/Cellulose Nanocrystal Composite Film

**DOI:** 10.3390/molecules28124667

**Published:** 2023-06-09

**Authors:** Tong Chen, Jun Li, Jun Xu, Yi Gao, Shiyun Zhu, Bin Wang, Guangdong Ying

**Affiliations:** 1 Plant Fiber Materials Research Center, State Key Laboratory of Pulp and Paper Engineering, South China University of Technology, Guangzhou 510640, China; 202020128116@mail.scut.edu.cn (T.C.); ppjunli@scut.edu.cn (J.L.); 18392415113@163.com (Y.G.); zhushiyun1992@scut.edu.cn (S.Z.); febwang@scut.edu.cn (B.W.); 2 Guangdong Provincial Key Laboratory of Plant Resources Biorefinery, Guangzhou 510006, China; 3 Shandong Sun Paper Industry Joint Stock Co., Ltd., Jining 272100, China; yinggdong@sunpaper.cn

**Keywords:** polylactide, cellulose nanocrystals, acetylation, tensile stress, compatibility

## Abstract

Polylactide (PLA) has become a popular alternative for petroleum-based plastics to reduce environmental pollution. The broader application of PLA is hampered by its brittle nature and incompatibility with the reinforcement phase. The aim of our work was to improve the ductility and compatibility of PLA composite film and investigate the mechanism by which nanocellulose enhances PLA polymer. Here, we present a robust PLA/nanocellulose hybrid film. Two different allomorphic cellulose nanocrystals (CNC-I and CNC-III) and their acetylated products (ACNC-I and ACNC-III) were used to realize better compatibility and mechanical performance in a hydrophobic PLA matrix. The tensile stress of the composite films with 3% ACNC-I and ACNC-III increased by 41.55% and 27.22% compared to pure PLA film, respectively. Compared to the CNC-I or CNC-III enhanced PLA composite films, the tensile stress of the films increased by 45.05% with 1% ACNC-I and 56.15% with 1% ACNC-III. In addition, PLA composite films with ACNCs showed better ductility and compatibility because the composite fracture gradually transitioned to a ductile fracture during the stretching process. As a result, ACNC-I and ACNC-III were found to be excellent reinforcing agents for the enhancement of the properties of polylactide composite film, and the replacement some petrochemical plastics with PLA composites would be very promising in actual life.

## 1. Introduction

Over the years, the extensive consumption of petroleum-based plastics has triggered serious environmental concerns because of their poor biodegradability. Around 80 billion kg of packing plastic derives from petroleum-based plastics each year, and most ends up in landfills or being incinerated [1]. However, landfills and incineration can contaminate soil and oceans and release toxic gas into the atmosphere, which can seriously damage the environmental equilibrium. Therefore, fully biodegradable material based on sustainable biomass is urgently needed. Recently, bio-plastics have aroused special interest because they can be synthesized from biomass resources and are more renewable and biodegradable than existing petroleum-based plastics [2,3]. The utilization of polylactic acid (PLA) is regarded as a promising sustainable approach to slow down the environmental impact from packing applications associated with the massive use of traditional plastics due to its outstanding physical properties and bio-based character [4,5,6]. Polylactic acid, a semicrystalline polymer with a glass transition temperature (T_g_) of about 60 °C and crystalline melting temperature (T_m_) of about 160 °C, has been used for diverse applications, such as plastic bags, food or medicine packing, portable tableware, and so on [7,8]. Compared with traditional petrochemical plastics, such as polypropylene (PP), low-density polyethylene (LDPE), polystyrene (PS), and nylon, PLA consumes 50% less non-renewable energy in the production process and can be completely degraded into water and carbon dioxide under natural conditions. However, PLA has not yet replaced conventional plastics due to its poor ductility, inherent brittleness, poor processability, and relatively high cost in practical usage [3,9,10,11]. In order to address these weaknesses and take full advantage of PLA, combining various nanoparticles with PLA polymer to form composite materials has been extensively studied [12,13,14,15].

Cellulose nanocrystals (CNCs) have been explored as a promising reinforcing phase in composite materials to improve stiffness and strength due to their excellent properties, such as high specific strength, low density, high aspect ratio, and low environmental impact [16,17,18,19]. CNCs, which are rod-like and highly crystalline nanometer-scale particles with dimensions of 5–20 nm in width and 20–500 nm in length, show improved mechanical properties; namely, the stiffness coefficient and tensile strength [20,21]. As a result, CNCs have been widely applied to reinforce PLA matrixes, but a mass of data has shown that the ductility and strength of the PLA-based materials remained unchanged or even decreased [22,23]. This could be attributed to the strong hydrophilic character and high aspect ratio of CNCs, which tend to flocculate, leading to poor interfacial interactions and compatibility between the hydrophilic additive and the hydrophobic PLA matrix [24,25,26,27]. Therefore, it would be significant to achieve a homogenous dispersion of CNCs in a polymeric matrix and further enhance the surface adhesion between the two polymers [4,28]. In order to expand the advantages of CNCs, several chemical modifications to decrease the surface hydrophilicity of CNCs have been reported, including esterification [29,30], silylation [31,32], oxidation [33,34], acetylation [35,36,37], and so forth. Among the potential chemical modifications that can be applied to decrease CNCs’ hydrophilicity, acetylation is regarded as a promising pathway to achieve tunable properties by only adjusting the acetyl content [28]. Cellulose is an allomorphic linear macromolecule including five types of polymorphs—namely, cellulose I, II, III, IV, and V—with different structures depending on the cellulose source, preparation process, and treatment. In general, cellulose I is a natural cellulose with parallel chains originating from numerous agricultural sources consisting of crystalline and amorphous regions [38,39]. Cellulose III is another common cellulose allomorph obtained by treating cellulose I or cellulose II with liquid ammonia or amines [40,41,42]. Moreover, it is a type of reactive crystalline cellulose that can used as a precursor in the production of various cellulose derivatives [43].

Although several reports have studied the properties of PLA reinforced with CNC-I, there is comparatively little research on PLA composites with the addition of CNC-III. It is noteworthy that the different arrangement of macromolecular chains in cellulose types I and III may result in differences in intramolecular and intermolecular forces, thus affecting the exposure of hydroxyl groups and their chemical reactivity. In our work, the aim incorporated the improvement of the interfacial compatibility between CNCs and the hydrophobic PLA matrix to develop well-dispersed and relatively renewable biomass nanocomposite films. In order to achieve this objective, two different polymorphs, CNC-I and CNC-III, were chemically modified using acetylation regents to reduce the hydrophilicity of the CNCs and further improve the polymer/nanoparticle interactions. Acetylation modification is used to transform the hydroxyl groups at C_6_ of cellulose into acetyl moieties and improve the dispersibility in PLA matrixes with low polarity. Therefore, PLA biomass composite films with improved properties, such as transparency, hydrophobicity, and mechanical performance, compared to unmodified CNCs were expected. In the present study, PLA biomass composite membranes with and without the addition of various quantities (1 wt%, 3 wt%, and 5 wt%) of CNC-I or CNC-III were prepared with a solvent casting approach. Moreover, the impacts of CNC dosage and acetylation modification of the two allomorphs on the properties of the PLA nanocomposites were also studied.

## 2. Results and Discussion

### 2.1. CNC and ACNC Characterization

#### 2.1.1. FT-IR and XRD Analysis

**Figure 1:**
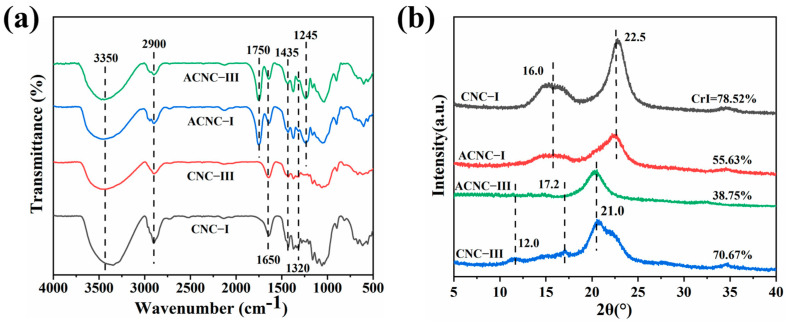
(**a**) FT-IR spectra of CNC-I, CNC-III, ACNC-I, and ACNC-III; (**b**) XRD spectra of CNC-I, CNC-III, ACNC-I, and ACNC-III.

Two cellulose nanocrystal polymorphs were evaluated by FT-IR. As shown in Figure 1a, it was clearly observed that all CNC specimens presented common adsorption bands at 3350 cm^−1^ and 2900 cm^−1^ corresponding to the O-H and C-H stretching peaks of the basic chemical structure of cellulose, respectively [44]. The intense peak at 1650 cm^−1^ in all curves was assigned to the O-H bending of the absorbed water because of the strong interaction between cellulose and water [45]. The new characteristic bands for ACNC-I and ACNC-III that appeared at 1750 cm^−1^ and 1245 cm^−1^ belonged to the C=O vibration of the ester groups and the C-O stretching vibration of carbonyl, respectively [46,47]. This demonstrated that the two cellulose nanocrystal polymorphs were successfully acetylated. The absorption bands located at 1435 and 1320 cm^−1^ were associated with the bending and wagging vibrations of C-H of cellulose [48].

The X-ray diffraction patterns of the CNC and ACNC allomorphs reflect the crystalline integrity of the CNCs. It can be seen from Figure 1b that the diffraction pattern of ACNC-I was similar to that of the unmodified CNC-I curve, showing no significant change, and they both displayed a sharp peak at 2θ = 16.0° and a weaker peak at 2θ = 22.5° corresponding to the (110) and (200) crystallographic planes, respectively [49,50]. This indicated that the main crystalline structure and allomorphy of CNC-I were not changed after acetylation. Similarly, for the XRD patterns of CNC-III and ACNC-III, the characteristic peaks at the 2θ angles of 12.0°, 17.2°, and 21.0° corresponded to the (010) and (002) planes and a complex of (110), (012), and (110) planes, respectively [51]. It is worth noting that the crystallinity of ACNC-I and ACNC-III decreased significantly after acetylation. Specifically, the decrystallization of ACNC-III was more obvious, which may have been due to the less compact crystalline structure of cellulose III [52].

#### 2.1.2. Contact Angle Analysis

**Figure 2:**
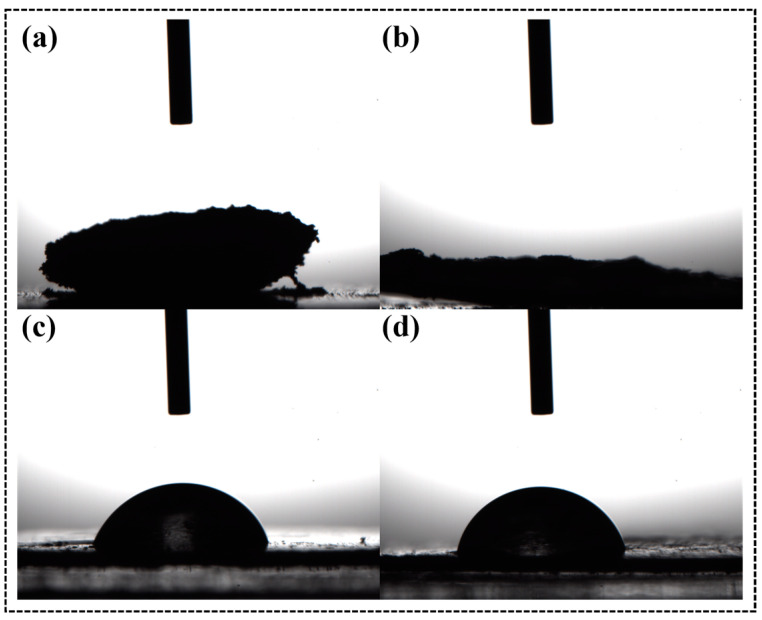
Contact angles of CNC and ACNC: (**a**) CNC-I; (**b**) CNC-III; (**c**) ACNC-I; (**d**) ACNC-III.

Figure 2 shows the contact angles of CNC-I, CNC-III, ACNC-I, and ACNC-III. The water droplets spread over the thin sheet made of unmodified CNC-I and CNC-III powder, which was attributed to the strong hydrophilicity of CNC-I and CNC-III. The contact angles of the acetylated ACNC-I and ACNC-III were 78.54 and 71.91, respectively, demonstrating that the acetylation could notably enhance the hydrophobicity property of the substances.

#### 2.1.3. Degree of Substitution (DS)

**Figure 3:**
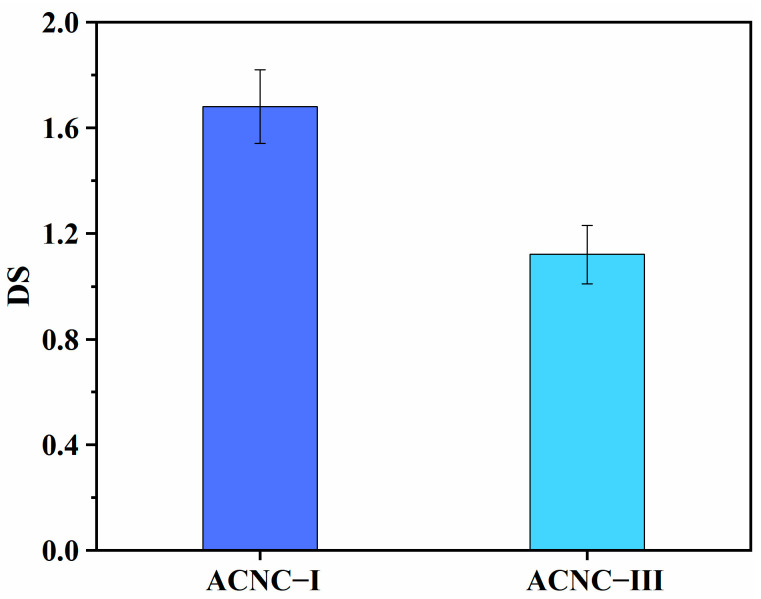
Degrees of substitution for ACNC-I and ACNC-III.

The degrees of substitution for the ACNC-I and ACNC-III samples are shown in Figure 3. It can be seen that the DSs of ACNC-I and ACNC-III were 1.68 and 1.12, respectively. Generally, the structure of CNC-III was looser and the accessibility of the chemical reaction of CNC-III should have been higher than that of CNC-I. However, the DS for CNC-III was lower than that of CNC-I, which was not consistent with the conclusions reported in the previous literature [41]. One reason for our results was that some flocculation inevitably occurred in the process of crystal conversion from CNC-I to CNC-III and the flocculation caused a reduction in the hydroxyl group exposed on the surface of the nanocellulose [38]. The lower hydroxyl contents resulted in fewer contact sites for acetylation reagents. Another more important reason was that the distance between its adjacent chains was shortened after cellulose I transformed into cellulose III, which increased the spatial resistance to mass transfer [52]. These reasons all led to lower accessibility and a lower degree of substitution for CNC-III in the acetylation process.

#### 2.1.4. Morphology Analysis of Nanocellulose CNCs

**Figure 4:**
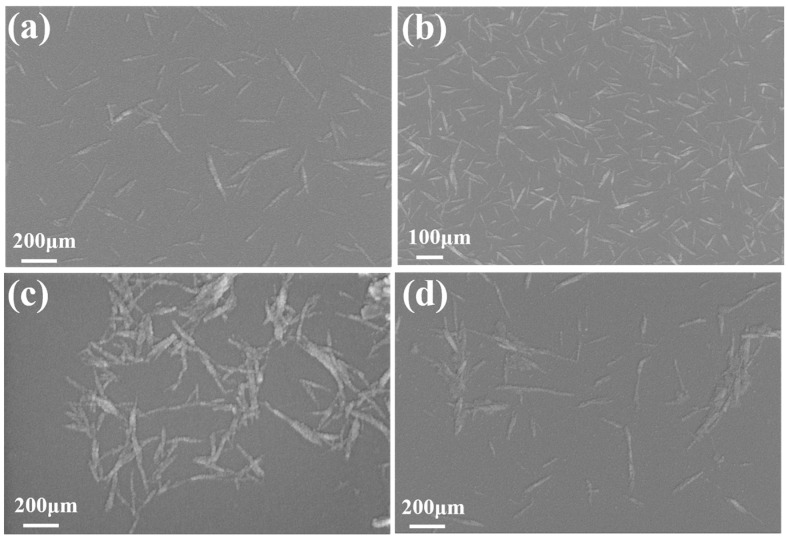
Scanning electron microscope images of CNCs and ACNCs: (**a**) CNC-I; (**b**) CNC-III; (**c**) ACNC-I; (**d**) ACNC-III.

As can be seen from Figure 4, CNC-III still maintained the same rod-like structure as CNC-I after transcrystallization. The length of CNC-III decreased from 160~260 nm to 150~200 nm as a result of transcrystallization. Figure 4c,d shows that ACNC-I and ACNC-III still retained the original rod-like morphology after acetylation, but the widths of ACNC-I and ACNC-III increased slightly. This indicated that the crystallization structures of ACNC-I and ACNC-III were eroded and moistened to a certain extent during the process of acetylation, thus increasing the widths of ACNC-I and ACNC-III.

### 2.2. Characterization of PLA/CNC and PLA/ACNC Composite Films

#### 2.2.1. Mechanical Properties Analysis

**Figure 5:**
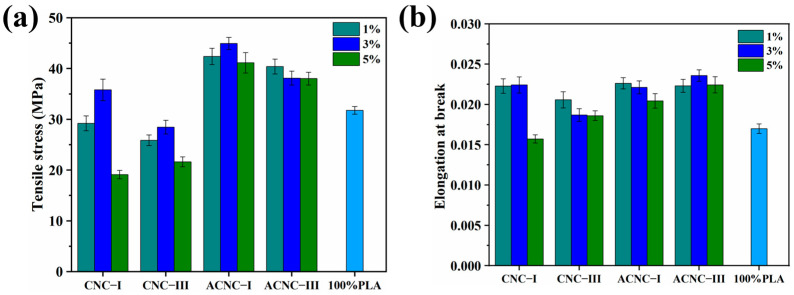
Mechanical properties of composite films: (**a**) histogram of tensile stress; (**b**) histogram of elongation at break.

Two mechanical properties, tensile strength and elongation at break, of the PLA/CNC composite films were investigated and the analysis results are shown in Figure 5. For the pure PLA film, the tensile strength and elongation at break were only 31.74 MPa and 1.17%, respectively. However, the composite films did not show much improvement in tensile properties compared to the pure PLA film when adding unmodified CNC-I and CNC-III, which was attributed to the presence of incompatible groups in the hydrophilic reinforcing agent and hydrophobic PLA molecules [53]. The incompatibility affected the hydrogen bonds and van der Waals forces and further destroyed the crosslinks between PLA molecular chains, resulting in worse mechanical performance for the composites. After acetylation, the surfaces of the ACNCs were grafted with non-polar groups, which relatively enhanced the surface hydrophobicity of the ACNCs and reduced the aggregation of the reinforcing agent in the PLA matrix. Generally, better compatibility between the reinforcing agent and polymer matrix had a positive effect on the mechanical properties of the composite film [54]. Hence, the tensile stress of the composite films with 1%, 3%, and 5% ACNC-I increased by 33.49%, 41.55%, and 29.55% compared to pure PLA films. The elongation at break of the composites with 1%, 3%, and 5% ACNC-I increased by 33.06%, 31.12%, and 20.29%. These results were also confirmed by TEM images (Figure S1a–d), which demonstrated the dispersion of CNCs and ACNCs in the PLA matrix. Some flocculation and large blank spaces can be seen in Figure S1a,b, which indicate the poor compatibility between CNC-I and CNC-III and PLA. The poor compatibility could hinder CNC-I and CNC-III from dispersing well in the PLA matrix and cause poor interface adhesion, thus leading to worse mechanical performance. Figure S1c,d show that the ACNC agglomerates were dispersed in a less compact manner and exhibited good interfacial adhesion to PLA. This was related to the better compatibility of the hydrophobic ACNC-I and ACNC-III in PLA [55].

Additionally, the impact of the cellulose nanocrystal polymorphs on the composite film with the same amount of reinforcing agent was investigated. The tensile stress of the CNC-I and ACNC-I composite films was greater than that of the corresponding composites prepared with CNC-III and ACNC-III. This could have been due to the shorter distance between adjacent molecules in CNC-III, resulting in fewer hydroxyl groups being exposed and a lower degree of acetylation. As a result, the poor compatibility between CNC-III and PLA caused by the lower degree of substitution in CNC-III had a negative effect on the mechanical properties [56]. At the same time, the stronger interchain binding force of CNC-III made it more difficult to disperse when it was compounded with PLA [28]. Therefore, the tensile stress of the composite film with 3% ACNC-III was only 27.22% higher than that of pure PLA film.

Furthermore, it can be observed that the CNC and ACNC content had a great impact on the mechanical performance of the membrane materials. The tensile stress and elongation at break of the composite materials were enhanced with increasing CNC content but decreased when the CNC content reached 3% or 5%. This means that the dispersion was better when the nanocrystals were added in a small amount as a result of the good mechanical performance of the nanocellulose itself. Nevertheless, with the increase in the CNC or ACNC content, self-agglomeration could occur in the PLA matrix, resulting in a larger filler size and smaller surface-area-to-volume ratio [57]. Furthermore, this could result in a negative influence on the interface adhesion and stress transfer between the PLA matrix and the fillers.

#### 2.2.2. Morphology Analysis of the Composite Film

**Figure 6:**
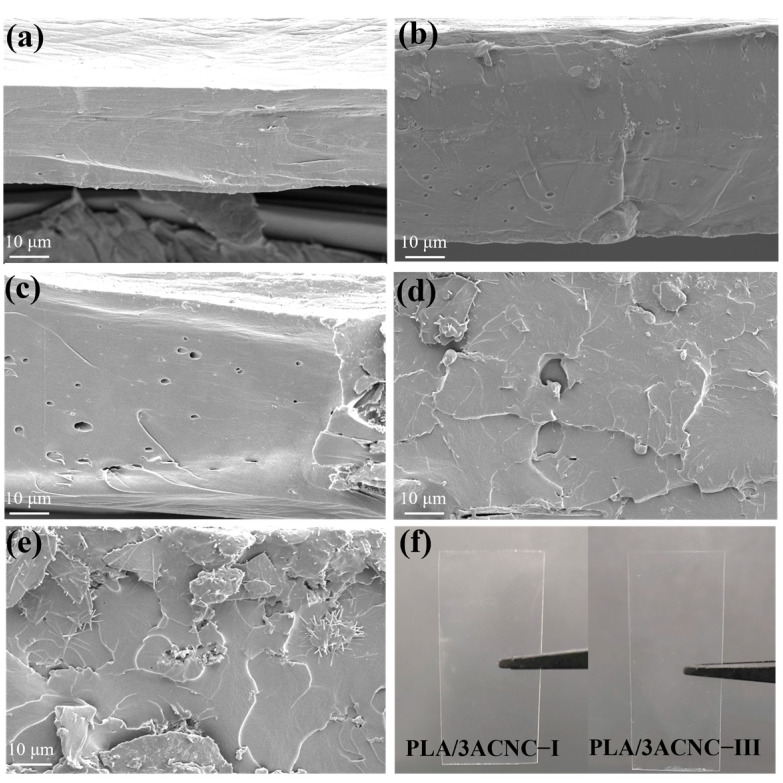
Scanning electron microscope images of tensile sections of PLA/CNC and PLA/ACNC composite films: (**a**) pure PLA film; (**b**) PLA/3CNC-I film; (**c**) PLA/3CNC-III film; (**d**) PLA/3ACNC-I film; (**e**) PLA/3ACNC-III film. (**f**) Macroscopic physical images of PLA/3CNC-I and PLA/3CNC-III composite films.

Figure 6a–e show the scanning electron microscopy images of the tensile fractured surfaces of the pure PLA and PLA bio-based composite films. It can be clearly observed from Figure 6a that the flat cross-section of the neat PLA displayed a quite smooth fractured surface and showed no obvious signs of tearing, which was typical and consistent with brittle polymer [58]. This was due to the high brittleness of PLA, and its fracture morphology belonged to the brittle fracture type. At the same time, taking into account the histogram of the elongation at break (Figure 5b), the pure PLA showed low elongation at break and low deformation caused by stretching, indicating that pure PLA possessed less ductility and greater brittleness. Figure 6b,c show the morphological characteristics of the PLA composite films with CNC-I and CNC-III; their sections presented several fracture traces because of the tensile force. In contrast, as shown in Figure 6d,e, the sections of the composite films with ACNC-I and ACNC-III presented more lamellar traces, which indicated that the PLA/ACNC composite films gradually transitioned to ductile fracture during the stretching process. These observations are further confirmed by Figure 6b, in which it can be seen that the elongation at break was higher than that of pure PLA film. Figure 6f shows macroscopic physical images of the PLA/3ACNC-I and PLA/3ACNC-III composite films. The two composite films could still maintain good light transmittance with the addition of ACNCs, which was in line with their use in daily life.

#### 2.2.3. Transmittance Analysis

**Figure 7:**
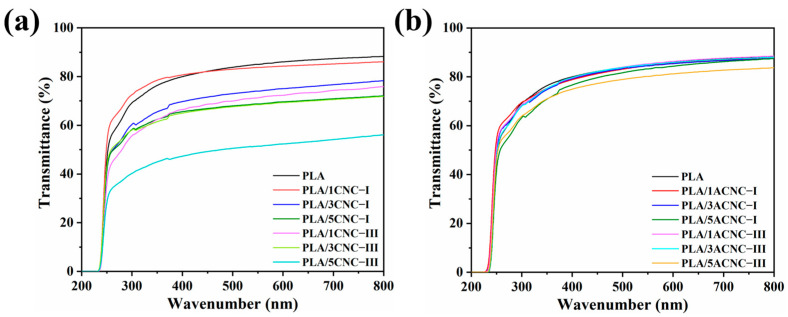
(**a**) Transmittances of PLA/CNC-I and PLA/CNC-III composite films; (**b**) transmittances of PLA/ACNC-I and PLA/ACNC-III composite films.

The acetylation effects of the two cellulose nanocrystal polymorphs (CNC-I and CNC-III) and their content on the transmittance of the composite film were further investigated. The transmittance of the films rose linearly at about 250 nm, then tended to remain stable at about 400 nm and, finally, stabilized between 50% and 90% at 700 nm (Figure 7a,b). The transmittance of PLA/1CNC-I was 85.19%, while the transmittance of PLA/1CNC-III was only 74.35% at 700 nm. Moreover, as shown in Figure 7a, pure PLA exhibited a high transmittance of 87.37%, while the composite film with 5% CNC-III showed the lowest transmittance of 54.16% at 700 nm. These results were due to the higher number of CNC-III agglomerations existing in the PLA matrix caused by the short distance between adjacent chains and the great spatial resistance to mass transfer of CNC-III. This indicated that the composite film employing CNC-III had a relatively better blocking effect against ultraviolet rays. Figure 7b shows that the transmittances of the composite films with low contents (1% and 3%) of ACNC-I or ACNC-III were all near 80%, and the type of polymorph and content of ACNCs had little effect on the transmittance of the composite films, which can be attributed to the great dispersion of the two types of acetylation-modified nanoparticles in the PLA matrix. The PLA/5CNC-I and PLA/5CNC-III composite films showed lower transmittance due to the self-agglomeration of high amounts of ACNCs dispersed in the PLA matrix [57].

#### 2.2.4. Thermal Stability Analysis

**Figure 8:**
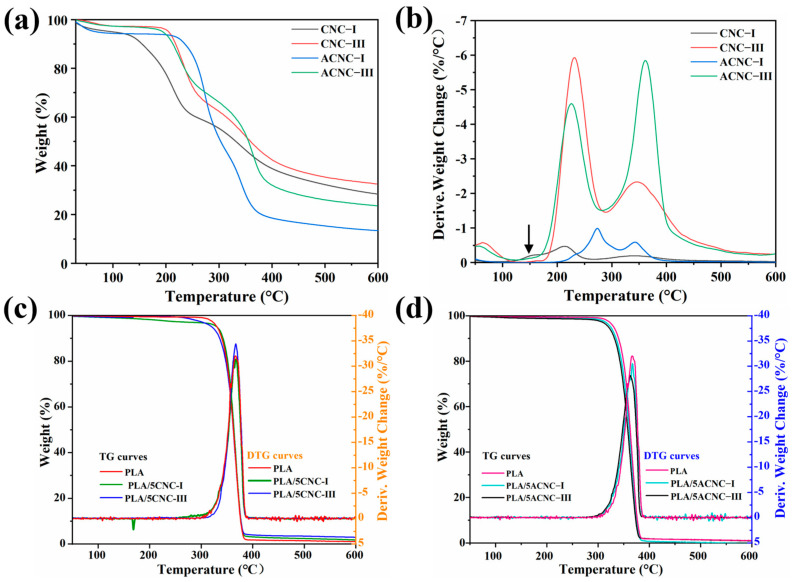
(**a**) TG curves for four kinds of nanocellulose; (**b**) DTG curves for four kinds of nanocellulose; (**c**) TG and DTG curves for PLA/CNC composite films; (**d**) TG and DTG curves for PLA/ACNC composite films.

The thermal stability and decomposition behavior of nanocellulose materials are significant for preparation and application of bio-based composites. The TG and DTG curves of four kinds of composite films—CNC, pure PLA, PLA/CNC, and PLA/ACNC films—are shown in Figure 8a–d. The degradation parameters of the composite films, including the onset decomposition temperature (*T_onset_*) and the maximum decomposition temperature (*T_max_*), are presented in Table 1. Figure 8a,b show that the degradation processes for all the CNC powders were different, but they basically had three stages of weightlessness. The first stage was assigned to the absorbed water of samples below 120 °C. The second stage occurred between 120 °C and 300 °C and corresponded to the thermal degradation of functional groups on the sample surface and the carbonization of cellulose. The third weightlessness stage was at about 300–500 °C and represented the slow carbonization of solid residuals [59]. The CNC-I film developed a shoulder peak at 165.7 °C corresponding to the catalytic degradation of the small amounts of sulfuric acid remaining on the sample surface [60]. However, the trace sulfuric acid remaining in the CNC-I sample and the sulfonic acid group on the surface of the CNC-I sample were neutralized with an alkaline swelling agent during the transition from CNC-I to CNC-III. Therefore, the shoulder peak at around 165 °C disappeared for CNC-III and the two *T_max_* peaks shifted to higher values. The *T_max_* values of CNC-III (211.9 °C and 343.2 °C) were higher than those for CNC-I (232.3 °C and 344.8 °C), and the *T_onset_* of CNC-III (190.8 °C) was also higher than that of CNC-I (130.2 °C). As we can see from Figure 8a,b, ACNC-I showed better thermal stability than CNC-I. The onset temperature increased from 130.2 °C to 216.8 °C due to the replacement of the original sulfate group with a more stable acetyl group [61,62]. However, ACNC-III displayed a different decomposition trend from ACNC-I. The *T_onset_* of ACNC-III was slightly lower than that of CNC-III, which was related to the lower crystallinity of CNC-III after crystal transformation and acetylation modification [38]. The TG and DTG curves for the PLA/CNC and PLA/ACNC composite films are presented in Figure 8c,d. Only a single degradation stage between 280 °C and 370 °C could be observed for the pure PLA and PLA/CNC bio-based composite films. Generally, the thermal stability of polymers can be enhanced by mixing them with nanofillers, and the dispersion condition of the nanofiller in the polymer matrix also ultimately affects the thermal properties of the composites. Surprisingly, the *T_onset_* of PLA/5CNC-I increased by more than 10 °C compared to the neat PLA film, indicating that the addition of CNC-I could effectively enhance the thermal performance of the composites. In comparison to CNC-I, the incorporation of ACNC-I, CNC-III, and ACNC-III showed no positive effects on the thermal stability of the composite films, which might have been related to their lower degree of crystallinity [38]. In general, the TG and DTG curves demonstrated that all the PLA composite films were thermally stable below 300 °C and could meet the processing and usage requirements.

## 3. Experiment

### 3.1. Materials

CNC-I samples were prepared from microcrystalline cellulose (MCC) using a conventional 64% sulfuric acid hydrolysis method at 45 °C for 45 min with a solid-to-liquid ratio of 1:10 [63]. CNC-III samples were obtained by impregnating CNC-I with ethylenediamine for 30 h [38]. Both CNC-I and CNC-III samples were freeze-dried prior to acetylation modification. MCC was acquired from Sigma-Aldrich, United States (St. Louis, MO, USA). Polylactic acid ((C_3_H_4_O_2_)_n_ (PLA)) was supplied by Dongguan Heshe Plastic Raw Material Co., Ltd. (Dongguan, China). Perchloric acid (HClO_4_) and phenolphthalein were acquired from Tianjin Oriental Chemical Factory (Tianjin, China). Ethanol was purchased from Sinopharm Chemical Reagent Co., Ltd. (Shanghai, China). Methylene dichloride (CH_2_Cl_2_), acetic acid (CH_3_COOH), acetic anhydride, (C_4_H_6_O_3_), and methylbenzene (C_7_H_8_) were all obtained from Guangzhou Chemical Reagent Factory (Guangzhou, China). Anhydrous ethylenediamine was provided by Shanghai Runjie Chemical Reagent Factory (Shanghai, China). Hydrochloric acid (HCl) and sodium hydroxide (NaOH) standard liquids at a concentration of 1 N were purchased from Shenzhen Bolinda Technology Co., Ltd. (Shenzhen, China).

### 3.2. Acetylation of CNC-I and CNC-III Samples

Briefly, CNC-I (0.2 g) and CNC-III (0.2 g) powders were added into methylbenzene solution (5 mL), respectively. The obtained mixtures were ultrasonicated by using an ultrasonic instrument (S-450D, BRANSON, American) at 40% power amplitude for 1 min in an ice bath. Then, acetic acid (4 mL), acetic anhydride (1.4 mL), and perchloric acid (0.02 mL) were poured into the above suspensions, and they were stirred continuously for 3 h at room temperature. The acetylation reaction was terminated by adding ethanol (20 mL). The obtained suspensions were dialyzed with ethanol for 3 days and deionized water for 7 days, respectively. All the acetylated samples were freeze-dried for 48 h to remove water and defined as ACNC-I and ACNC-III for subsequent usage.

### 3.3. Preparation of the PLA/ACNC Composite Films

PLA/ACNC-I and PLA/ACNC-III composite films were prepared using a solution casting approach [64]. The 0.02, 0.06, and 0.10 g ACNC-I and ACNC-III samples were dispersed in methylene dichloride (30 mL), respectively, and mixed for 5 min with ultrasonic treatment. Subsequently, 1.98, 1.94, and 1.90 g PLA were added into the above suspensions, and stirring was continued until clear solutions were obtained. In particular, the total weight of the PLA and CNCs or ACNCs was set at 2 g to ensure the same quantity of film. After defoaming in a vacuum environment, the mixtures were poured into a square glass bottle. Finally, the PLA/ACNC composite films were obtained by allowing the solvent to naturally evaporate overnight. The PLA/ACNC composite films were designated as PLA/1ACNC-I, PLA/3ACNC-I, PLA/5ACNC-I, PLA/1ACNC-III, PLA/3ACNC-III, and PLA/5ACNC-III, respectively, in terms of the addition of ACNCs (1 wt%, 3 wt%, and 5 wt%) to the PLA. Moreover, in accordance with the above operations, PLA/CNC composite films were prepared in the same way as the control group.

### 3.4. Characterization of CNCs and ACNCs

An FT-IR spectrometer (VERTEX 70, Bruker, Mannheim, Germany) was used to detect the functional groups of the two cellulose nanocrystal polymorphs before and after modification. The resolution was set to 4 cm^−1^ and the range to 4000–400 cm^−1^ for scans. X-ray diffraction measurement of the CNC and ACNC samples was performed using an X-ray generator (X^’^Pert^3^, PANalytical B. V, Almelo, The Netherlands) with a scanning rate of 4° min^−1^ at 40 mA and 40 kV with Ni-filtered Cu Kα radiation in the angular range of 5–80°. A scanning electron microscope (SEM) (Merlin, Zeiss, Oberkochen, Germany) was used to observe the morphological changes in the nanocellulose after modification at an operating voltage of 5 kV.

The contact angle test was used to measure the effect of acetylation on the hydrophobicity of the CNC-I and CNC-III samples. It was carried out with an optical contact angle measuring instrument (CA100, Beidou instrument, Chongqing, China) and the data recording time was 10 s. The CNC-I, CNC-III, ACNC-I, and CNC-III powders were pressed into thin sheets mechanically before the contact angle test.

The acetyl content and the degree of substitution (DS) for the ACNC-I and ACNC-III samples were tested using heterogeneous saponification and back-titration with HCl [65]. Dried ACNC-I or ACNC-III (0.1 g) samples were transferred to a 150 mL iodine flask containing ethanol (10 mL), which was then sealed and heated at 50 °C for 30 min. Then, a few drops of 0.1 N NaOH were added to the above suspension to obtain a permanent pink color using phenolphthalein as an alkaline indicator. Afterwards, NaOH (10 mL, 0.1 N) was added to the flask and it was heated at 50 °C for 30 min to full saponification. The flask was then left to react at room temperature for 72 h. After the reaction, the remaining NaOH present in the iodine flasks was back-titrated with 0.1 N HCl until the solution changed from being pink to colorless. Moreover, the unmodified CNC-I and CNC-III samples were used for blank titration and the volume of HCl consumed was recorded as *V*_0_. The acetyl content (%) and the degree of substitution were calculated with the following equations:

(1) $$Acetylcontent\left(\%\right)=\frac{V_{0}{-V}_{1}\times N\times 0.043\times 100}{W}$$

where *V*_0_ is the volume of 0.1 N HCl consumed by the blank sample, and *V*_1_ is the volume of 0.1 N HCl consumed by the acetylated ACNC-I or ACNC-III. N is the normality of 0.1 N HCl. W is the mass of the ACNC sample used.

(2) $$DS=\frac{162\times A}{4300-42A}$$

where *A* is the acetyl content.

### 3.5. Characterization of PLA/CNC and PLA/ACNC Composite Films

A transmission electron microscope (TEM) (JEM-1400F, JEOL, Tokyo, Japan) was employed to observe the plane morphology of the specimens at an accelerating voltage of 100 KV. The preparation of the TEM test samples was as follows: the composite film was cut into thin slices with a frozen ultra-thin slicer (EM UC6+FC6, Leica, Wetzlar, Germany), and then the slices were placed on the copper net of the transmission electron microscope for photographing after natural air drying. A scanning electron microscope (SEM) (Merlin, Zeiss, Germany) was used to detect the cross-sectional structure of the PLA composite film at an operating voltage of 5 kV. A UV spectrophotometer (UV2600, Shimadzu Instrument, Kyoto, Japan) was used for transmittance detection and the test wavelength range was 200–800 nm. The mechanical properties of the composite film were measured using a universal testing machine (INSTRON 5565, Norwood, MA, USA) according to the Chinese standard GB/T 1040.3-2006. The stretching velocity was 5 mm min^−1^ and at least five replicates were employed for each membrane. Tensile strength and elongation at break were detected and analyzed from the stress–strain curves. Thermal gravimetric analysis (TGA) was carried out with a thermogravimetric analyzer (TG209 F1 LIBRA, NETASCH, Bayern, Germany). Approximately 5–10 mg of sample, dried at 60 °C for 5 h prior to detection, was heated from room temperature to 600 °C at a rate of 10 °C min^−1^ under a nitrogen flow of 20 mL min^−1^.

## 4. Conclusions

In conclusion, this study highlights the benefits of using two nanocellulose polymorphs and their acetylated products as functional additives to reinforce PLA composites. To enhance the compatibility between the CNCs and the PLA matrix, CNC-I and CNC-III powders were acetylated and then used as the reinforcing phase for PLA. The PLA/ACNC-I and PLA/ACNC-III composite films exhibited better surface adhesion and mechanical properties than those of the PLA/CNC-I and PLA/CNC-III composite films, allowing for practical usage in packing material. The improvement in ductility and dispersibility demonstrated the satisfactory compatibility between the ACNCs and the PLA matrix. Specifically, the enhancement effect from the ACNC-I sample was generally higher than that from the ACNC-III sample due to the differing structural performance of the two CNC polymorphs. The optimal mechanical properties for the composite film were obtained with 3% ACNC-I loading. The optimal value for the tensile strength was 1.42 times that of the pure PLA film and 1.26 times that of the PLA/CNC-I composite film, respectively. In general, this study is able to provide a straightforward method for designing PLA composite films with required properties by combining different amounts of CNC and ACNC polymorphs.

## Figures and Tables

**Table 1:** Degradation temperatures of CNCs and their composite films.

Specimen	T_onset_ (°C)	T_max_ (°C)		
CNC-I	130.2	165.7	211.9	343.2
CNC-III	190.8		232.3	344.8
ACNC-I	216.8		273.6	343.8
ACNC-III	185.8		228.3	363.3
PLA	308.5		366.1	
PLA/CNC-I-5%	318.9		366.8	
PLA/CNC-III-5%	303.2		366.6	
PLA/ACNC-I-5%	302.9		366.8	
PLA/ACNC-III-5%	300.7		362.9	

## Data Availability

The data presented in this study are available on request from the corresponding author.

## Supplementary Materials

The following supporting information can be downloaded at: https://www.mdpi.com/article/10.3390/molecules28124667/s1. Figure S1. TEM images of PLA/CNC and PLA/ACNC composite films: (a) PLA/3CNC-I; (b) PLA/3CNC-III; (c) PLA/3ACNC-I; (d) PLA/3ACNC-III.

## Sample Availability

Samples of the compounds are available from the authors.
