# Supplementary material for: Effect of Acetylation of Two Cellulose Nanocrystal Polymorphs on Processibility and Physical Properties of Polylactide/Cellulose Nanocrystal Composite Film
Source: Molecules. 2023 Jun 9;28(12):4667. doi: 10.3390/molecules28124667 (PMC10302014; doi:10.3390/molecules28124667)
Supplement: Supplementary file 1 [file molecules-28-04667-s001.zip › molecules-2419718-supplementary.pdf]

Effect of Acetylation of Two Cellulose Nanocrystal Polymorphs on Processibility and Physical Properties of Polylactide/Cellulose Nanocrystal Composite Film

Tong Chen [1], Jun Li [1], Jun Xu [1,2,*], Yi Gao [1], Shiyun Zhu [1], Bin Wang [1] and Guangdong Ying [3]

[1] Plant Fiber Materials Research Center, State Key Laboratory of Pulp and Paper Engineering, South China University of Technology, Guangzhou 510640, China; 202020128116@mail.scut.edu.cn (T.C.); ppjunli@scut.edu.cn (J.L.); 18392415113@163.com (Y.G.); zhushiyun1992@scut.edu.cn (S.Z.); febwang@scut.edu.cn (B.W.)

[2] Guangdong Provincial Key Laboratory of Plant Resources Biorefinery, Guangzhou 510006, China

[3] Shandong Sun Paper Industry Joint Stock Co., Ltd., Jining 272100, China; yinggdong@sunpaper.cn

* Correspondence: xujun@scut.edu.cn

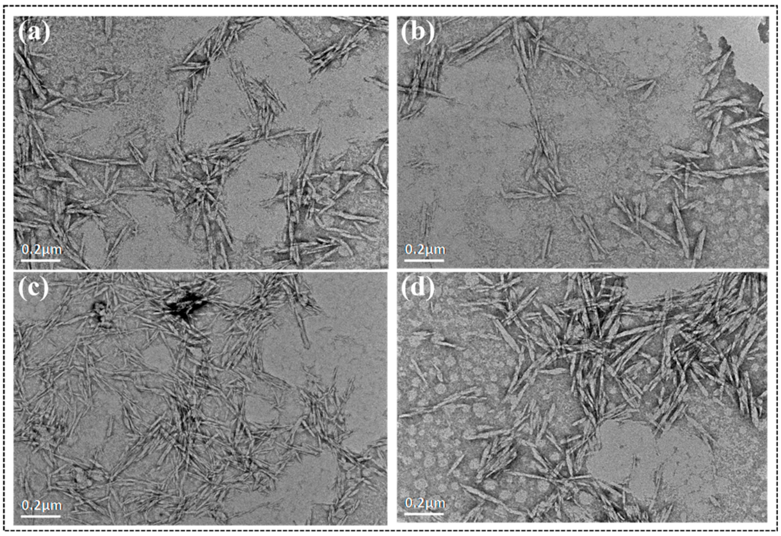

Figure S1. TEM images of PLA/CNC and PLA/ACNC composite films: (a) PLA/3CNC-I; (b) PLA/3CNC-III; (c) PLA/3ACNC-I; (d) PLA/3ACNC-III.
